# Incorporating clinical guidelines through clinician decision-making

**DOI:** 10.1186/1748-5908-3-13

**Published:** 2008-02-29

**Authors:** Paul R Falzer, Brent A Moore, D Melissa Garman

**Affiliations:** 1 Clinical Epidemiology Research Center, VA Connecticut Healthcare System, West Haven, CT, USA; 2 Department of Psychiatry, Yale School of Medicine, New Haven, CT, USA; 3 Southwest Mental Health System, Connecticut Department of Mental Health and Addiction Services, Bridgeport, CT, USA

## Abstract

**Background:**

It is generally acknowledged that a disparity between knowledge and its implementation is adversely affecting quality of care. An example commonly cited is the failure of clinicians to follow clinical guidelines. A guiding assumption of this view is that adherence should be gauged by a standard of conformance. At least some guideline developers dispute this assumption and claim that their efforts are intended to inform and assist clinical practice, not to function as standards of performance. However, their ability to assist and inform will remain limited until an alternative to the conformance criterion is proposed that gauges how evidence-based guidelines are incorporated into clinical decisions.

**Methods:**

The proposed investigation has two specific aims to identify the processes that affect decisions about incorporating clinical guidelines, and then to develop ad test a strategy that promotes the utilization of evidence-based practices. This paper focuses on the first aim. It presents the rationale, introduces the clinical paradigm of treatment-resistant schizophrenia, and discusses an exemplar of clinician non-conformance to a clinical guideline. A modification of the original study is proposed that targets psychiatric trainees and draws on a cognitively rich theory of decision-making to formulate hypotheses about how the guideline is incorporated into treatment decisions. Twenty volunteer subjects recruited from an accredited psychiatry training program will respond to sixty-four vignettes that represent a fully crossed 2 × 2 × 2 × 4 within-subjects design. The variables consist of criteria contained in the clinical guideline and other relevant factors. Subjects will also respond to a subset of eight vignettes that assesses their overall impression of the guideline. Generalization estimating equation models will be used to test the study's principal hypothesis and perform secondary analyses.

**Implications:**

The original design of phase two of the proposed investigation will be changed in recognition of newly published literature on the relative effectiveness of treatments for schizophrenia. It is suggested that this literature supports the notion that guidelines serve a valuable function as decision tools, and substantiates the importance of decision-making as the means by which general principles are incorporated into clinical practice.

## Introduction

In their report on translating behavioral science into action, The National Advisory Mental Health Council notes: 'At present too few researchers are attempting to bridge across basic, clinical, and services research, and not enough are working with colleagues in related allied disciplines to move research advances out of the laboratory and into clinical care, service delivery, and policymaking [1].

What has resulted from this situation might be called the 'dissemination gap' – a disparity between knowledge and its implementation [2]. A number of investigators have emphasized that disjunctions between research and application [3-5], science and service [6,7], and research and policy [8] are adversely affecting the quality of public mental health programs and services. Though such problems have been appreciated since at least the late 1940s, the current effort to bridge clinical research and practice began in earnest in the early 1990s, with the telling words of Congressman George Brown: 'All the basic science funding in the world will have no positive effect on the well-being of our nation if the research is not carried out within a system that can effectively digest and apply the results' [9].

The dissemination gap is amply illustrated by complications of implementing evidence-based practices (EBPs) in the medical branch of psychiatry. Most psychiatric practitioners acknowledge that clinical decisions and practices should be based on the best available evidence [10]. Nonetheless, EBPs have not been received with the enthusiasm that was anticipated, and a variety of explanations have been proposed for the apparent anomaly. Even a cursory review of the literature shows that the implementation of EBPs is a polarizing issue. One group of researchers tout their importance for quality of care and their relevance to mental health policy [11]. Others retort by warning practitioners to 'proceed with care!' [12]. They are concerned about a potentially deleterious effect of EBPs on quality of care [13] and believe that the implementation of EBPs is on a collision course with the movement to individualize care and promote patient-centered healthcare [14,15].

One of the most frequently expressed reasons for the failure to adopt EBPs in medicine, particularly psychiatry and mental health, pertains to the decision-making practices of clinicians. Studies have shown that lack of adherence to EBPs is a result of systemic decisional biases [16,17]. These claims can draw support from hundreds of studies conducted over the past fifty years. Beginning with Meehl's [18] research that compared clinical with actuarial prediction, and proceeding through the work of Goldberg [19], Dawes [20], and others, studies have found almost universally that clinicians lack the consistency and the perceptive and integrative power that are requisite to sound decision-making. In short, they tend be 'suboptimal' decision-makers who, as such, perform less capably than statistical models [21,22].

Perhaps, as Proctor suggests, such broad-based conclusions do little more than identify easy targets and offer simplistic answers [23]. Nonetheless, lack of endorsement for EBPs, well-documented resistance on the part of some clinicians, and their tendencies toward sub-optimal decision-making lead to three related conclusions: Sound clinical judgment is a predicate of evidence-based medicine; there is a need for more research on how the implementation of EBPs is affected by clinical decision-making, and medical education should give priority to training clinicians in how to make good clinical decisions [24-26]. To lead the effort to understand more fully that factors that influence the uptake of EBPs and encourage their broader dissemination, the National Institute of Mental Health recently advanced a series of organizational and programmatic initiatives intended to expand the frontier of implementation research and raise the profile of dissemination [27]. The proposed study responds to this call.

A useful way to understand the dissemination problem is through the lens of evidence-based medicine (EBM), which Sackett and associates define as: 'the conscientious, explicit, and judicious use of current best evidence in making decisions about the care of individual patients' [28]. It may be tempting to regard EBM as the mere implementation of policies and procedures, then to gauge their effectiveness by how closely they are followed in clinical practice. In fact, the relationship between EBM and EBPs is far richer, and their relevance to clinicians is considerably more complex: EBM uses EBPs to aid clinical judgment. EBPs and clinical guidelines must be employed conscientiously and incorporated judiciously. For example, the American Psychiatric Association's practice guideline development process depicts guidelines as 'strategies to assist psychiatrists in clinical decision-making' [29]. Consider as well the opening statement of the APA's schizophrenia guidelines: 'This report is not intended to be construed or to serve as a standard of medical care' [30].

'Incorporation' is the key concept in the preceding passages. Well-intentioned efforts that equate conformance to a guideline with its utilization in practice have tended to exacerbate the very resistance that EBM was seeking to overcome [31]. Battle lines are drawn when clinicians are told, in one way or another, that the quality of their work can be gauged by how closely they conform to guidelines that were developed for the express purpose of aiding their decisions rather than directing them [32,33]. Despite the beneficent objective of improving quality of care, such efforts tend to polarize and backfire. The study described here attempts to break this impasse by proposing incorporation rather than conformance as the principal test of utilization, and developing a strategy that is designed to encourage clinicians to incorporate EBPs into their practice. As noted above, guidelines and EBPs are incorporated into through decision-making. As Patel and associates contend, 'evidence is invariably perceived evidence in the physicians' mind' [[34], p. 162], and the diffusion of EBPs will lead to greater utilization insofar as they fit the cognitive processes of decision-makers [35-37].

This proposed investigation has two specific aims: to identify the processes that affect decisions about incorporating clinical guidelines, and then to develop and test a strategy that promotes the utilization of EBPs. This paper focuses on the first aim and proceeds in three steps: First, it describes a preliminary piece of research showing that psychiatrists fail to adhere to a specific evidence-based guideline that they are acquainted with and endorse in principle. Second, it discusses how the appropriate use of decision theory can facilitate a richer understanding of these findings. The third step is modify the procedure employed previously and conduct an empirical investigation of how the guideline is incorporated into treatment decisions. This paper describes the proposed methodology, including procedures, hypotheses, and the plan for data analysis. Plans for components two and three, including the need for modifications that have come to light subsequent to the protocol's development, are described in the discussion section of this paper.

## Background and significance

### Evidence-based practices in psychiatry

It is possible to conduct a general examination of evidence-based practices and their implementation, but not without overlooking the specialization of knowledge that pervades medical training, research, and practice. In psychiatry, every major disorder – in some cases, multiple subcategories within disorders – have distinct practices and guidelines. The proposed study focuses on the clinical paradigm of schizophrenia, a severe mental illness that afflicts over two million Americans [38]. The cause of schizophrenia has yet to be definitively identified and aspects of its nature remain mysterious. Nonetheless, significant gains have been made, especially over the past twenty years, in developing effective symptomatic treatments [39]. Along with comprehensive approaches to service delivery [40], current treatments enable patients to live independently in the community as they recover from the illness [41,42].

However, there is a subset consisting of 10 to 30% of patients who have an inadequate response to antipsychotic medications [30,43]. They comprise the category known as treatment-resistant schizophrenia (TRS). Studies have examined the effectiveness of various antipsychotic medications for TRS, including clozapine [44,45] and other second generation antipsychotics (SGA's, also known as 'atypical antipsychotics'), either individually or in combination [46-48]. By and large, these treatments have demonstrated limited effectiveness, but given the lack of more efficacious alternatives they nonetheless are valuable components of the clinical armamentarium. Although there is little dispute about the importance of identifying patients who have treatment resistant disorders, there are no universally accepted classification criteria. In general, investigators have adapted criteria initially advanced by Kane [49], of positive symptoms (delusions, hallucinations, disorganization) that are not significantly reduced or adequately remediated following two full trials of antipsychotic medication. Overall, difficulties of classification, overall lack of treatment effectiveness, and lack of consensus over the best course of treatment make TRS a complex and vexing clinical situation and an attractive paradigm for studying the role of clinical guidelines in treatment decision-making.

The general problem of implementing EBPs in the treatment of schizophrenia was amply illustrated by the response to the Schizophrenia Patient Outcomes Research Team (PORT) treatment guidelines. These guidelines were developed from a review of the literature by experts and widely disseminated in 1998 [50]. Buchanan [51] examined the records of 224 inpatients and 368 outpatients with schizophrenia and found a high rate (greater than 95 percent) of conformance with PORT's medication guidelines, but low conformance with dosage recommendations (62 percent for inpatients and 28 percent for outpatients). These findings were consistent with a previous finding about medications being used ineffectively, either at levels below therapeutic dosage or above maximum recommend dosage [52].

The original PORT guidelines were revised in 2004 [53], largely in response to the growing literature on the relative efficacy of first- versus second-generation antipsychotic medications (FGAs versus SGAs). Despite the initial enthusiasm that accompanied the approval and release of SGAs such as risperidone, olanzapine, and ziprasidone, as a group they appear not to be more effective, at least for initial or first-break treatment, than the considerably less expensive FGA's such as haloperidol, perphenazine, and fluphenazine [54]. Consequently, revised PORT recommended FGAs as the first line of treatment. In addition, several studies had demonstrated that clozapine, the prototypical SGA, was particularly effective for treatment resistant schizophrenia [44,55]. The revised PORT guideline recommended clozapine treatment for TRS, despite the difficulties of managing the medication and its potentially severe side effects [56].

Using the revised PORT guidelines as a template, Sernyak and associates [57] developed an evidence-based guideline for TRS, then tested clinician conformance, using clinicians who had been introduced to the guideline, understood its use, and explicitly concurred with it. The TRS guideline developed by Sernyak *et al*. is summarized in Table 1. It calls for patients to be assessed at the end of a three-month medication trial, using the Clinical Global Inventory (CGI) to assess progress and illness severity [58]. A 'non-responder' was defined as a patient whose CGI scores indicate at least a moderate mental illness, and a condition that has either not improved or has deteriorated over the course of treatment. Adhering to the guideline involves moving one step up the treatment ladder, beginning another three-month trial, assessing again, then continuing up the ladder until the patient no longer meets non-responder criteria. Sernyak and associates examined the charts of the prescribing physician subjects. Almost half of their patients with schizophrenia met the TRS criteria, but for 95 percent of these patients, the physician elected to maintain the current medication in lieu of following the TRS guideline.

**Table 1:** The Guideline for the Treatment Resistant Schizophrenia

**Step**	**Treatment**	**Common Examples**
1	FGA #1: 1st trial of a first-generation (conventional) antipsychotic medication	haloperidol (Haldol), perphenazine (Trilafan), fluphenazine (Prolixin), loxapine (Loxitane), molindone (Moban), thiothixene (Navane) trifluoperazine (Stelazine)
2	FGA #2: 2nd trial of a first-generation antipsychotic medication	as above
3	SGA #1: 1st trial of a second-generation (atypical) antipsychotic medication	risperidone (Risperdal), aripiprazole (Abilify), quetiapine (Seroquel), olanzapine (Zyprexa), serlect (Sertindole)
4	SGA #2: 2nd trial of a second-generation antipsychotic medication	as above
5	Clozapine	clozapine (Clozaril) only

The findings obtained by Sernyak and associates were both surprising and expected. On the one hand, they are consistent with other studies about conformance with the PORT schizophrenia guidelines [59]. Reasons for departing from the guideline included the patient's unwillingness to change the current medication, non-adherence to medications, a current condition of being stable, or the clinician's judgment that more time is needed with the current regimen. These reasons are similar to the items identified in the latest Schizophrenia PORT guidelines as factors to be considered in choosing an antipsychotic medication [53]. On the other hand, it is puzzling that in 95 percent of cases failed to conform to the TRS guideline when clinicians were knowledgeable of the guideline and concurred with it in principle.

The proposed study examines the apparent anomaly in how clinicians use the TRS guideline by employing three principal modifications of the procedure employed by Sernyak and associates: First, instead of consulting clinical charts, the proposed study uses case vignettes, which permit a precise manipulation of the factors that influence treatment decisions, hypothesis testing about their relative importance, and a descriptive account of decision strategies.

Second, whereas the Sernyak study used both psychiatric residents and staff psychiatrists as subjects, the current study focuses on psychiatric trainees – third-, fourth-, and fifth-year residents who have experience in treating adults with disorders in the schizophrenia spectrum. This approach enables the investigation to identify one specific group of clinicians where training can have a discernible effect on decision-making. Studies of pattern learning and clinical judgment have found that experts and trainees use different patterns of reasoning [60,61], and that these differences affect their use of clinical guidelines [35]. These findings suggest that even if an experienced psychiatrist and a resident make the same treatment decision in a given case, they will go about the task differently. One of the limitations of previous implementation efforts lies in their failure to tailor the intervention to a specific audience and facilitate its dissemination.

Third, the proposed study draws expressly on the tradition of behavioral decision-making to describe the role of the TRS guideline in clinicians' decisional processes. The following section discusses this tradition and indicates why a naturalistic approach to decision-making and a cognitively-rich naturalistic theory has been chosen over the more customary models that are derived from classical decision theory.

### Decision-making and evidence-based practices

Behavioral decision theory has played a significant role in medical research and education, and the importance of decision-making for clinical practice has long been recognized [62-64]. Unfortunately, much of this work has relied on models of classical decision theory (CDT), which arguably have limited application to the task of understanding how clinicians make treatment decisions, particularly under practical constraints such as having limited information, working with complex disorders that have heterogeneous outcomes, and prescribing for patients who have treatment-resistant conditions. The principal problem with CDT is its all-consuming interest in making optimal decisions, which putatively result from the proper employment of a logical model. The normative thrust of CDT was evident from its origin in the work of Pascal [65], to the initial forays into behavioral decision-making by von Neumann and Morgenstern [66], and later, through models developed by experimental and social psychologists that have been applied directly to clinical practice [67,68]. Elstein [69] makes an explicit link between EBM and CDT by asserting that a treatment is 'evidence-based' when it has a higher probability of achieving a desirable outcome than a non-evidence-based alternative. The guiding notion is that the best clinical outcome will be attained by choosing the intervention that maximizes expected value.

These findings and the procedures that led to them have been questioned on several grounds [70-74]. For one, the idea of comparing actual with optimal decisions enables investigators to observe how frequently decision-makers fall short of an ideal, but a prescriptive focus does not suffice as an explanation of how, where, and why decision-making goes wrong. This limitation of normative models is particularly important in clinical education and research, where the objective is to correct and improve decision-making, not merely observe that it has fallen short. Moreover, comparing actual practices against guidelines implicitly construes the latter as standards of optimality and involves a test of conformance. It does not enable investigators to determine whether guidelines have been incorporated into clinical decision processes. The difference is particularly important, given that clinicians can conform to clinical guidelines without understanding why they should be applied. Concomitantly, an appropriate use of guidelines is to take them into account without following them in a particular case.

A number of investigators have expressed dissatisfaction with the inveterate use of normative modeling and attempted to predicate behavioral decision theory on more empirical and descriptive grounds. A host of exemplary adaptations of CDT have followed, some of which focus specifically on the decision-making practices of physicians and other clinicians [75-78]. The best known exemplar is 'cognitive heuristics,' which Kahneman and Tversky developed from a series of clever and compelling studies demonstrating that decision-making is suboptimal because people simply do not follow normative strategies [79,80]. This work has won a prominent place in the decision-making literature, particularly the study of medical decision-making [62,68,81,82]. However, a premise of this approach is that cognitive short-cuts inevitably lead to inaccurate judgments, a notion that has been vigorously challenged in a variety of studies, including applications of clinical decision-making [83,84]. Moreover, the notion that decision-makers function as transducers, and employ a cognitive apparatus that works like a choice-selecting mechanism, is not a desirable or reasonable way of depicting clinical practice. When decisions are made with limited information, uncertainties are incalculable, and there is no universally correct answer, cognition plays a role more akin to active problem solving than passive optimizing.

### The naturalistic alternative

Some decision researchers contended that CDT should not be repaired, but supplanted by empirically-based theories, and proceeded to develop the approach known as Naturalistic Decision-Making (NDM) [85]. The application of NDM to medicine was actually pioneered by Elstein [61,86], who decried the surfeit of anecdotes that have been inspired by accounts of suboptimal decision-making and the gratuitous recommendations of what clinicians ought to be doing. The wisdom inspired by CDT presumes a friction-free environment that fails to appreciate the concrete situations that decision-makers confront, particularly when they ae working with complex clinical problems.

Patel and associates built on Elstein's work in several ways. For them, the persistent gap between knowledge and its implementation is a result of failing to understand how information contributes to knowledge, and how the gap between knowledge and information can be addressed by focusing on the cognitive processes of makers [34,35]. These investigators, along with other proponents of a cognitive-rich naturalistic approach to clinical decision-making, are suspicious of initiatives 'that promote blind adherence to a course of action without targeting the understanding in any meaningful sense' [73].

One of the most prolific advocates of NDM in cognitive and organizational psychology is Lee Beach, who began working on a naturalistic alternative after devoting his early career to the study of classical theory. In addition to issuing a protracted critique of CDT [87], Beach developed Image Theory (IT), which is one of the most fully articulated and extensively researched of all the NDM-inspired theories [87-89]. A distinctive feature of IT is that it posits that decision-making involves the simple application of multiple strategies, in contrast to CDT, which posits that decision-making involves complex applications of a single strategy.

For IT, decision processes begin with winnowing or filtering out unacceptable alternatives, and only if necessary proceeding to a second stage of choosing a preferred alternative [90,91]. The mechanism of discarding unacceptable alternatives is called screening. Much of the time, decisions are made simply or principally by excluding alternatives that are incompatible with internally-generated criteria. For example, the choice of where to eat lunch is based on proximity, cost, health, and taste. Restaurants are excluded or 'screened out' that are too far away, too expensive, or serve food that is unhealthy or unpleasant. Each instance of a disjunction between attribute and criterion counts as a 'violation', and options are discarded whose violation count exceeds an arbitrary critical number known as the 'rejection threshold' [92,93]. In the example above, one may reject restaurants that fail at least two attributes, but those that fail none or only one remain as candidates for a subsequent selection procedure.

Two distinctive characteristics of screening concern the weighting of attributes and the consistency of the rejection threshold. IT studies have shown that the most common screening strategy involves unit-weighting [92,94]. In that event, alternatives are excluded simply by the violation count: If the four criteria of restaurants are regarded as equally important, then those whose violations exceed the rejection threshold are screened out, regardless of which specific criteria are violated. With differential weighting, certain violations are regarded as more important than others. For instance, proximity may have twice the value of cost, unhealthiness may be three times as important as proximity, etc. [94].

Findings from IT research indicate that screening and choice are distinct decision strategies that occur in sequence. If more than one candidate survives the screening stage, a choice will be made invoking a more exhaustive procedure. Identical criteria can be utilized in both phases, but studies have shown that they are weighed and aggregated differently [95]. Commonly, screening eliminates all candidates except one and therefore becomes determinative. For instance, consider that most treatment decisions are binomial: Providers either continue the current treatment or switch to another therapy. If we assume that this decision is made by means of a choice strategy, then it would involve an exhaustive comparison of alternative treatments. If instead, the decision is to continue current treatment unless there are specific reasons to reject it – or if the decision is to follow a treatment guideline unless there are specific reasons not to – then screening is the definitive decision strategy.

It is suggested that clinicians incorporate EBPs into their decisional processes through the mechanism of screening. Instead of, or at least prior to, their using a guideline to assist in making a choice, they assess the guideline's applicability to the case at hand by using pertinent clinical facts as screening criteria. Thus, when the clinicians in the Sernyak study reported reasons for departing from the guideline that included the patient's unwillingness to change the current medication, non-adherence, the stability of the current condition, or the need for more time to evaluate treatment response, they were saying, in effect: I will follow the TRS guideline unless there is sufficient reason not to follow it. I may determine that the guideline is not appropriate for a given case. If so, my decision in this instance does not represent a wholesale rejection. Even if the guideline does not survive to the end of my decisional process, it played a crucial role at the beginning. The proposed study examines whether indeed psychiatric residents are applying a screening strategy to the TRS guideline; if so, whether they are weighing violations equally, following a consistent rejection threshold, or operating in a more incremental fashion.

## Methods

### Study population, subjects, and recruitment

Twenty volunteer subjects will be recruited from the population of third-, fourth-, and fifth-year residents in an accredited psychiatry training program who are currently treating or have recently treated adults who have schizophrenia or schizo-affective disorder. The study will not use first-and second-year residents, who either lack sufficient knowledge about diagnosis and treatment to make independent clinical decisions, or lack experience in working with a severely mentally ill population. Given the institutional affiliation of the principal investigator and the funding source, study protocols and consent forms will be approved by three Human Investigation Committees.

The protocols call for subjects to be recruited passively through general announcements, advertisements in common resident areas, through the department listserv, and by word-of-mouth. It is essential that participation be regarded as entirely voluntary and minimize the possibility that candidates might feel pressure to participate. Efforts will be made to ensure that participation is neither encouraged nor required by the residency program, that no one associated with the program would see individual study data, and that the study would not be used to assess their progress. The study task requires about one hour and subjects will be paid $100 for participating.

### Procedures, instruments, and design

Subjects will be handed a loose leaf binder containing the two consent forms, a registration form, general information about treatment-resistant schizophrenia and the TRS guideline, and the study stimulus. Subjects can consult this information at any time. The stimulus consists of 72 vignettes that pertain to a hypothetical 24 year old male with schizophrenia. The vignettes will be presented sequentially in three groups: The first 32 pertain to step two of the guideline; the second 32 pertain to step four, and the other eight are designed to test the subject's expectation about the general effectiveness of the guideline. The same set of vignettes appear in both step two and step four. The final eight are drawn systematically from the 32, as described below. The order of items in each of the three sets will be randomized into four stimulus groups (in no case will the order of the vignettes in steps two and four correspond). Subjects will receive one of the four groups at random.

The 32 vignettes for steps two and four represent all combinations of a 2 × 2 × 2 × 4 design:

### Progress

Two levels, representing a score of four or six on the Clinical Global Inventory (CGI, the instrument used to assess treatment resistance). A score of six indicates a lack of sufficient progress and is a criterion of treatment resistance. A score of four indicates sufficient progress to disqualify the patient from moving to the next step.

### Condition

Two levels, representing a score of three or seven on the CGI, where seven indicates an unacceptable low level of functioning and a criterion of treatment resistance, and three indicates sufficient progress to disqualify the patient from moving to the next step.

### Adherence to antipsychotic medications

Two levels, operationally defined as a rate of 25% (low adherence) or 75% (high adherence).

### Progress forecast

Four expected outcomes if the guideline were to be followed in this case:

Ineffective: 10% likelihood of significant improvement, 80% likelihood of no significant change, and 10% likelihood of decompensation;

High risk: 45% likelihood of significant improvement, 10% likelihood of no change, 45% likelihood of decompensation;

Low gain: 10% likelihood of significant improvement, 40% likelihood of no change, 50% likelihood of decompensation.

High gain: 50% likelihood significant improvement, 40% likelihood of no change, 10% likelihood of decompensation.

### Violations

For the purpose of the study, a violation is a clinical factor that is either inconsistent with the guideline or a reason to depart from it. The lower scores in factors one and two above indicate that the patient is responding sufficiently to current treatment and does not meet the TRS criteria. Low adherence and poor forecasted progress do not appear in the guideline, but nonetheless serve as potentially powerful reasons not to recommend a medication change. A good forecast is high gain (forecast four, above); ineffective, high risk, or low gain forecasts are all regarded as poor. For the 32 vignettes, one had no violations, six vignettes had one, twelve had two, ten had three, and three vignettes had four violations. The subset of eight vignettes are used to inquire about step three of the TRS guideline and consist of factors two and three, and forecasts three and four. (The progress factor will be held constant at six, which is not a violation of the guideline). Of the eight vignettes, one has 0 violations, three vignettes have one, three have two violations, and one vignette has three.

### Data collection, hypotheses, and data analysis

For the 32 case vignettes at steps two and four of the TRS guideline, subjects will indicate whether they would proceed to the next step of the guideline or not, then rate their confidence in the decision on a seven-point scale that ranges from extremely confident to not confident at all. For the eight vignettes at step three, subjects will make three ratings, each as a percentage from zero to 100: the likelihood that following the protocol will ever result in a good treatment response; the likelihood that the patient will ever have a good response by not following the protocol, and the likelihood that the clinician would follow the step four of the guideline after an insufficient response at step three. Data will also be collected on subject gender, age, race, ethnicity, and year in the residency program.

Hypotheses will be tested using the General Estimating Equation (GEE) algorithm in SPSS version 15 [96]. GEE models are appropriate for measuring the magnitude of association for dichotomous correlated outcomes, which commonly are encountered in repeated-measures or within-subjects designs [97]. GEE models have become popular in mental health services research, owing to the importance of acknowledging interdependence of criterion variables, as well as greater availability of statistical software [98,99] GEE permits models to be examined for overall significance and for the significance of individuals main effects and interaction, using the Wald test [100].

### Principal Hypothesis: Subjects incorporate the TRS guideline into their treatment decisions via a unit-weighted screening strategy

In a screening strategy, the number of violations is determinative and specific violations, both individual and in relationship to one another, are irrelevant. With unit-weighting, each violation is equally important. Thus, the study hypothesis is that a greater number violations is associated with a greater likelihood of rejection. We will test whether a summary score containing the total number of violations predicts rejection of the guideline. Follow-up analysis will evaluate whether there is evidence of a 'rejection threshold' – an hypothesized point at which violations exceed an arbitrary standard. For this analysis we will compare, Bonferroni protected, each sum of violations against zero violations. This analysis will be followed by testing each sum against the level immediately below it (two violations versus one, three violations versus two, four violations versus three). The pattern of significant and non-significant effects will provide information on where the rejection threshold(s) occurs. Unit-weighting predicts that each of the four violations – progress, condition, adherence, and forecast – will have a similar effect on outcome. For the second test of the unit-weighting hypothesis, we will evaluate the impact of each violation based on the rejection threshold. That is, if the rejection threshold is one, each violation type should be significantly associated with rejection. If the rejection threshold is two, all two-violation combinations should significantly predict rejection.

### Secondary analyses

As this study is exploratory, it relies on descriptive and post-hoc analyses to inform the investigators about the processes that effect subjects' decisions to endorse or reject the TRS guideline. Consequently, we will examine the influence of the individual factors that are manipulated in the study design. The general strategy is to begin by focusing on the three variables – 'step,' 'forecast,' and 'adherence' – that are not TRS guideline criteria, but nonetheless may constitute screening strategy violations. Following these analyses, we will inquire into whether – and if so, how – subjects endorse the guideline by examining the two TRS criteria, 'progress' and 'condition.'

Secondary analyses will also evaluate alternatives to unit weighting, including non-unit weighted screening, contingency models [101], and Recognition Primed Decision-making [102,103]. The latter two approaches are applications of naturalistic decision-making that may be particularly useful if subjects endorse the guideline under certain conditions and not others, or if there are identifiable subsets of subjects who are more inclined than others to endorse the guideline. Secondary analyses will create models that evaluate the violations as main effects and interactions and compare treatment decisions with confidence assessments. Since these analyses are exploratory, we will rely on Pan's extensions of Akaike's information criterion (AIC) test [104] to compare models for goodness-of-fit.

## Discussion

It is anticipated that proposed study will affirm the value of examining how cognitive processes of clinicians effect their treatment decisions. In addition, it may lead to broader application of an incorporation test for clinical guidelines. Its first application is in developing a strategy that increases the implementation of evidence-based practices by assisting clinicians in how to make complex treatment decisions. This approach may prove more effective than adherence-based dissemination programs or procedures that may be more appropriate for classic cases than for what clinicians commonly encounter in consulting rooms, medication clinics, and community treatment facilities.

Originally, the study called for phase two to duplicate the methods described here, using different variables. Significant developments that occurred after the project was conceived and funded have led to modifying the original plan. The TRS guideline has had a limited shelf life, and been supplanted by another guideline [105]. Meanwhile, one of the key assumptions of both the old and new guidelines is that FGAs should be first-line treatments. Recent studies have called this assumption into question [39,106,107]. After careful consideration, it was decided that phase two will use the Texas Medication Algorithm, Schizophrenia Module (TMA) [108], a guideline that differs from TRS in several respects. Besides using SGAs as first-line treatments, TMA calls for earlier implementation of clozapine therapy and the use of adjunctive treatments. It contains a validated measure of progress and employs three categories of response – full, partial, and non-response [109]. The latter feature is particularly important because it enables phase two to examine partial responses, which require clinician discretion and are more conducive to a standard of incorporation than adherence.

Changes in guidelines for the treatment of schizophrenia, particularly TRS, were inevitable, given the sweep of events that has followed completion of the first phase of the Clinical Antipsychotic Trials of Intervention Effectiveness (CATIE) study [110]. CATIE offered a head-to-head comparison of FGAs and SGAs against a variety of treatment outcome measures. Results to date found shown only modest differences, a high dropout rate, and rather limited effectiveness overall. The study's Principal Investigator reflected on the findings and expressed the following opinion:

'The CATIE results suggest that first-generation antipsychotics remain useful and deserve continued consideration by clinicians and patients. This does not mean that older, cheaper antipsychotics can replace more expensive second-generation agents. It is crucial to point out that equivalent does not mean identical: 25 percent of patients may respond to risperidone and 25 percent to perphenazine, but they are not the same 25 percent. These initial results from CATIE speak to the need for treatment options – not restrictions, such as closed formularies or fail-first requirements' [111].

If we construed guidelines as rules or performance standards, Lieberman's statement could be interpreted as rejecting their application to the treatment of schizophrenia because there is an insufficient evidence base. Perhaps instead, his reflection is a call to better assist clinicians in making difficult decisions, precisely because the available evidence is not determinative. If the latter interpretation has merit, then Lieberman is affirming the value of assisting physicians in how to use clinical guidelines without following them blindly.

Services and implementation research have tended to equate guidelines with EBPs, but this equation may be premature if not misleading. At this stage of our knowledge, particularly about severe mental illness, the two remain distinct and appreciating their relationship may be important to developing effective implementation strategies. In the first place, guidelines serve multiple masters. There is the evidence, of course. But several options that are available any given decision point may qualify as 'evidence-based.' Which option is recommended may depend on factors such as cost, ease of administration, effectiveness with the greatest number of patients, and amenability to adjunctive or combination therapies.

Ironically, guidelines about implementation of evidence-based practices may never have been scrutinized for effectiveness or subjected to randomized trials. On some occasions, guidelines are invoked to assist clinicians in choosing among competing evidence-based treatment options; at other times, they enable clinicians to veer must off the clinical path and prescribe treatments in a manner that has never been explicitly tested. For example, step four of the TRS guideline calls for selection of a second SGA; this choice is in lieu of clozapine, which is the medication of choice at step five. Even though the efficacy of second generation antipsychotic medications has been well established by randomized control trials, their effectiveness has not been demonstrated specifically for decisions at step four of the TRS guideline – when patients have not responded adequately to two trials of FGAs and one trial of an SGA.

Despite its sensibility and its likeness to the PORT schizophrenia guidelines, the TRS guideline is a novel application that moves beyond the boundary of established and demonstrably effective treatment. In this regard, TRS is not the rule and not an exception. Expecting clinicians to follow it, or the recommendations of a similar guideline, automatically is tantamount to poor practice. But if the guideline is incorporated into a decision strategy, it can become a valuable means of transcending the dissemination gap and improving quality of care. Developing and testing such a strategy is the principal task of phase two and the subject of a separate report.

## Competing interests

Paul R. Falzer is principal investigator on the NIMH-funded study reported here, Decision-making and the Dissemination of Evidence-based Practices (R34-MH070871-01A2).

Brent A. Moore, is the principal investigator on an NIMH-funded study, Selegiline for Treatment of Cannabis Dependence (R21 DA019246-01). The authors have no competing interests.

## Authors' contributions

PRF conceived the study, wrote the funding application, and serves as the study PI. BAM is a co-investigator and the study's methodologist. DMG is the consulting investigator and the study's clinical consultant. All three authors contributed to writing and editing the manuscript.
